# Different memory patterns of digits: a functional MRI study

**DOI:** 10.1186/s12929-019-0516-y

**Published:** 2019-03-04

**Authors:** Jingxin Nie, Zengqiang Zhang, Bin Wang, Hong Li, Jianghua Xu, Sheng Wu, Chunhua Zhu, Xin Yang, Bin Liu, Yongming Wu, Sheng Tan, Zhibo Wen, Jinlong Zheng, Siyun Shu, Lin Ma

**Affiliations:** 1 School of Psychology, Center for Studies of Psychological Application, South China Normal University, Guangzhou, 510631 China; 2 Department of Neurology, Zhujiang Hospital, Southern Medical University, Guangzhou, 510282 China; 3 Pediatric Center, Zhujiang Hospital, Southern Medical University, Guangzhou, 510282 China; 4 Hangzhou Sanatorium of air force, 15th Yanggongdi Road, Hangzhou, 310007 China; 5 Hangzhou Sanatorium of Army, 27 Yang-gong Di, Hangzhou, 310007 China; 6 The first Sanatorium of PLA Navy, Qingdao, 266071 China; 7 Department of Emergency, Zhujiang Hospital, Southern Medical University, Guangzhou, 510282 China; 8 Department of Neurology, Nanfang Hospital, Southern Medical University, Guangzhou, 510515 China; 9 Department of Radiology, Zhujiang Hospital, Southern Medical University, Guangzhou, 510282 China; 10 Department of Neurology, Huai’an First People’s Hospital, Nanjing Medical University, Huai’an Jiangsu, 223300 China; 11 Department of Radiology, The General Hospital of Chinese People’s Liberation Army, Bejing, 100853 China

**Keywords:** Memory for numeric figures, Short-term memory, Long-term memory, Working memory, Striatum

## Abstract

**Background:**

Psychological investigations and functional imaging technology have been used to describe neural correlations of different types of memory with various stimuli. Memory with limited storage capacity and a short retention time can be classified as short-term memory (STM) while long-term memory (LTM) can be life-long without defined capacity.

**Methods:**

To identify brain activation pattern associated with different modes of memory for numerical figures, we detected brain activities from twenty-two healthy subjects when performing three types of memory tasks for numbers, namely STM, LTM and working memory (WM), by using functional magnetic resonance imaging (fMRI) technique.

**Results:**

The result revealed variable patterns of activation in different brain regions responding to different types of memory tasks. The activation regions with primary processing and transient maintenance of STM for numerical figures are located in the visual cortex and mainly encoded by visual representations, while LTM was encoded by semantics and mainly recruiting left frontal cortex. We also found that subcortical structures, such as the caudate nucleus and the marginal division of the striatum, plays important roles in working memory.

**Conclusions:**

Activation of different brain regions in these three kinds of memories, indicating that different kinds of memories rely on different neural correlates and mental processes.

## Introduction

Memory is one of the most essential abilities of the human brain. According to the stage model theory [1], the memory system can be divided into three independent sub-systems, including sensory memory, short-term memory (STM) and long-term memory (LTM), while working memory (WM) is not included. WM refers to the ability of transient storage and manipulation of information held activated for further usage in related cognitive processes or for goal-directed behavioral guidance [2]. These three kinds of memory are highly interconnected so that they cannot work independently in any simple cognition task. WM needs to process novel information from STM and also input/retrieve information to/from LTM contiguously. It means WM is continuously playing some roles in complex cognitive tasks by connecting STM and LTM [3, 4]. For example, during the course of calculation, STM is engaged to store the individual digits and their locations within the number, and WM retrieves arithmetic knowledge (addition or multiplication table) from LTM to manipulate the numbers in a specific order [5]. Digits are special and flexible, with various levels of representational properties [6]. They are used for counting items, telling the time, calculating prices, identifying telephone numbers, keeping scores of sport games, and so on. Digits (numbers) are derived from languages and feature a complex representational system in the different area of the brain.

**Fig. 1:**
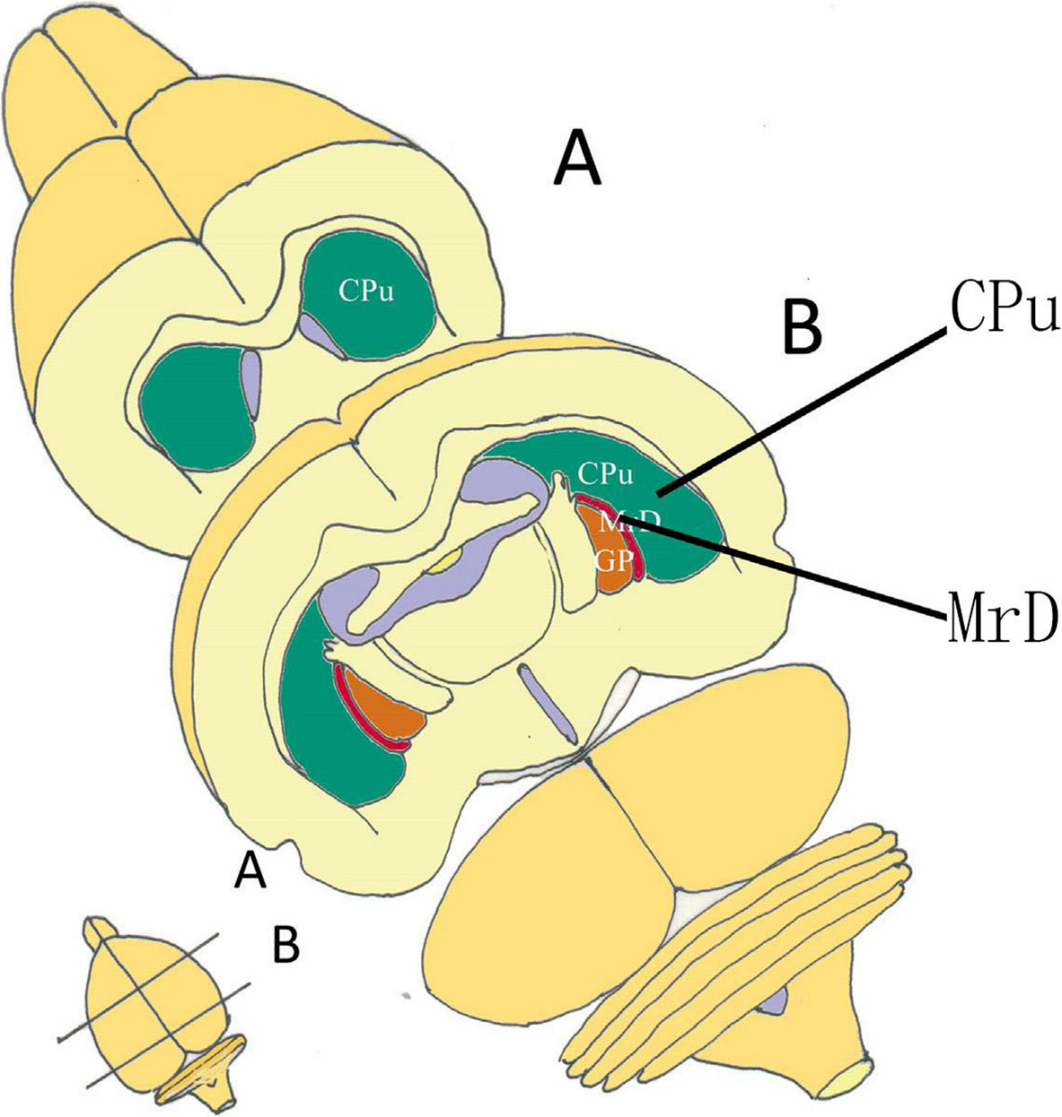
The figure shows the location and cytoarchitectural characteristics of the MrD in the rat brain. The schemes on the left side represent frontal section pictures of the rat brain (shown in the lower left corner). The MrD is at the caudomedial border of the CPu and rostrolateral to the globus pallidus

A newly discovered subdivision consisted of fusiform neurons in the ventromedial margin of the striatum in the brains of the rat, cat, monkey and human was discovered and termed “the marginal division (MrD) of the striatum” (Fig. 1). A variety of neuropeptides and receptors were found intensely expressing in the fusiform neurons of the MrD [7, 8]. The pedunculopontine nucleus gave rise to massive afferent terminals in the MrD of the squirrel monkey [9]. The MrD connected to the interstitial nucleus of the posterior limb of the anterior commissure [10]. The a2- adrenergic receptors were more highly expressed in the MrD than the rest of the rat striatum [11]. The MrD was verified to play important role in learning and memory by Y-maze test, long-term potentiation and patch clamp in rats [12–14]. The function of the MrD was identified with functional magnetic resonance imaging (fMRI) of healthy volunteers tested with an auditory digital WM task. Highly active areas were observed in the prefrontal cortex and MrD with left sided predominance during performance of the task [15]. A unique case provides clinical evidence that the medial area, including the MrD, of left putamen might play a critical role in learning and memory in human brain. These findings support the importance of imaging of the medial part of the putamen in patients complaining of memory deficits not explained by alternative etiologies [16].

Dehaene proposed the triple-code model for mental calculation, which hypothesized that different brain regions were responsible for processing spoken numbers, recalling numerical knowledge, calculation and comparing magnitudes respectively [17–19]. To verify this hypothesis, task-related components (for instance, magnitudes representation or numerical knowledge) were found with activities in bilateral inferior parietal, left perisylvian, and ventral occipitotemporal areas in an fMRI study [20]. Short-term maintenance processes have been found to be associated mainly with areas in the ventrolateral prefrontal cortex (VLPFC, Brodmann (BA) 44/45/47), the dorsolateral prefrontal cortex (DLPFC, BA 8/9/46), and posterior parietal cortex [21, 22]. Unlike STM, LTM has a relatively large capacity and much longer maintenance, and is acquired by repeated learning new information processed in WM. The functional neuroimaging studies on WM indicated that the prefrontal cortex was important for WM [23, 24]. Learning and memory deficits were caused by a lesion in the marginal of the left putamen in the human brain [16]. With functional MRI (fMRI) techniques, studies revealed that MrD and prefrontal cortex were involved in digital WM in human brain [15, 25]. The processes and mechanism of learning and memory involved in the MrD and hippocampus may be different [26]. The functional connectivity between the MrD and the identified regions was significantly correlated with the neuropsychological scores among the mild cognitive impairment (MCI) and Alzheimer’s disease (AD) subjects. The MrD functional network is disrupted during AD [27]. The findings suggested that the structure measurements based on corrected phase images and diffusion tensor imaging could provide a simple and effective tool for the evaluation of MrD in vivo in the human brain and for the assessment of the changes seen with aging [28]. Different views on neural substrates for digital memory have also been reported in different studies [29, 30]. However, it is still not well understood how the human brain memorizes digits.

Since Arabic numbers are special characters within complex representational systems, the questions as to how the human brain stores and processes them have been raised. To identify the neural substrates recruited for STM and LTM as well as WM for digits, we administered digit sequence trials while fMRI scans were performed on the subjects at the same time in this study.

## Methods

### Subjects

Twenty-two healthy subjects (13 males and 9 females, aged 21.45 ± 1.37 years old) participated in the present study. All subjects were Chinese-speaking undergraduate students of Southern Medical College of China and right-handed according to the Edinburgh handedness inventory [31]. The study protocol was approved by the Institute Review Board of South China Normal University. Written informed consent complied with the Declaration of Helsinki (1975) was obtained from each subject. All of these subjects were free from psychiatric or neurological illness as assessed by a psychiatrist, and none of them was under any medication or had any history of head injury.

### Tasks

Three fMRI experiments were performed in blocks with visual stimuli. Each experimental cycle consisted of four phases and each of which contains one memory block and one control block. Memory block and control block were performed alternatively and the whole cycle lasted for 4 min. All the trials consisted of visually presented Arabic digits, and the subjects were asked to press the right-hand button to respond accordingly. All introduction and guidance words were presented in simplified Chinese. The detail of these three experiments are described as follows.

## Short-term memory for numeric figures

Seven single-digit numbers were used as stimuli according to the average chunking capacity for seven digits [32]. Mathematically structured numbers (for example, 8,421,248) were eliminated to avoid the strategic encoding effect on the lateral prefrontal cortex [33]. During the experiment, after the word “Start” was shown on the screen for 1.5 s, a series of 7 Arabic numerical figures of single digit (ranged from 0 to 9, named memory sample) was shown in a random order at the center of the screen at the speed of 0.75 s per figure followed by the word “Remember” shown on the screen for 1 s, and then another series of 7 numeric figures (named probe) was shown in the same way at the same speed. After these two series of numeric figures, the instruction “Judge” was displayed on the screen for 2 s for the subjects to determine whether the probe matched (digits and their order) the memory sample or not. The subjects were asked to press a button if the two series were the same with their right hand and do nothing if they were not the same. Hence each memory trials took 15 s (0.75 s × 7 × 2 + 2 s + 1.5 s + 1 s). Two memory trials were repeated in each block. In the control trials, a numeric figure (ranged from 0 to 9) was shown on the screen for 13 s, and then the question “Is it 5?” appeared on the screen. The subjects were then asked to give an answer in 2 s by pressing the right-hand button for yes and doing nothing for no. Control trial was repeated twice in each block.

## Long-term memory for numerical figures

In this experiment, subjects were given eighteen double-digit numbers to remember 3 days prior to the experiment. During the test, after the word “Start” was shown on the screen for 2 s, thirteen double-digit numerical figures were shown on the screen in turn at the speed of 2 s per figure. The subjects were asked to press the button with their right hand immediately when they thought that the displayed figure was one of the figures that were given to remember before and do nothing if not. In control trial, thirteen double-digit figures were shown on the screen in turn at the speed of 2 s per figure. When the 13 double-digit figures were shown in turn, the subjects were asked to determine whether currently displayed figure is “50” or not. The subjects were asked to press the button with their right hand if the figure “50” were shown.

## Working memory for numerical figures

After the word “Start” was shown on the screen for 2 s, thirteen numerical figures of double-digit were displayed at the speed of 2 s per figure, and then a 2 s interval following the 13 figures. During the test each subject was asked to press the right-hand button when he or she thought that the displayed figure had ever appeared at least once before in this trial. In the control trial, thirteen double-digit figures were shown on the screen in turn at the speed of 2 s per figure. When the 13 double-digit figures were shown in turn, the subjects were asked to determine whether currently displayed figure is “50” or not. The subjects were asked to press the button with their right hand if the figure “50” were shown.

### MRI data acquisition

The fMRI data were acquired from a Siemens Sonata 1.5 T MR scanner. For each subject, we acquired brain functional images by using a single-shot gradient-echo EPI sequence. With the following parameters, echo time (TE) = 49 ms, repetition time (TR) = 3000 ms, flip angle = 90°, field of view (FoV) = 210 mm × 210 mm, data matrix = 64 × 64 matrix, slice thickness = 5 mm, 30 slices were acquired every 3.0 s. Eight dummy scans were performed prior to the image acquisition to eliminate signals arising from progressive saturation. High-resolution (1.2 mm × 1.2 mm × 5 mm) T1 structural images were also acquired for each subject.

### Data analyses

The fMRI data were processed using SPM (Statistics parametric mapping, Department of Cognitive Neurology; http://www.fil.ion.ucl.ac.uk/spm). Images obtained from the first 6 s of each acquisition session were removed from further functional data processing to minimize the transit effects of hemodynamic responses. The preprocessing steps includes the slice-timing correction, realignment, co-registration, normalization and smoothing. For each individual, the fMRI data from subjects, whose head motion in spatial translation was over 1 mm or head rotation was over 1° in any direction, were removed from the further data analyses. Activated brain maps were generated using a temporal-correlation method, in which the BOLD signal in a voxel was correlated to a boxcar function that was convolved with the canonical hemodynamic response.

Subject-specific linear contrasts, including the encoding versus baseline condition and the retrieval versus baseline condition for each of the effects of interest, were assessed. These contrasts were entered into a standard SPM second-level analysis, treating subjects as a random effect for one-sample *t*-test. A voxelwise intensity threshold (*P* < 0.001) and a spatial extent (5 voxels as the minimum cluster size) were set for multiple comparisons.

## Results

**Fig. 2:**
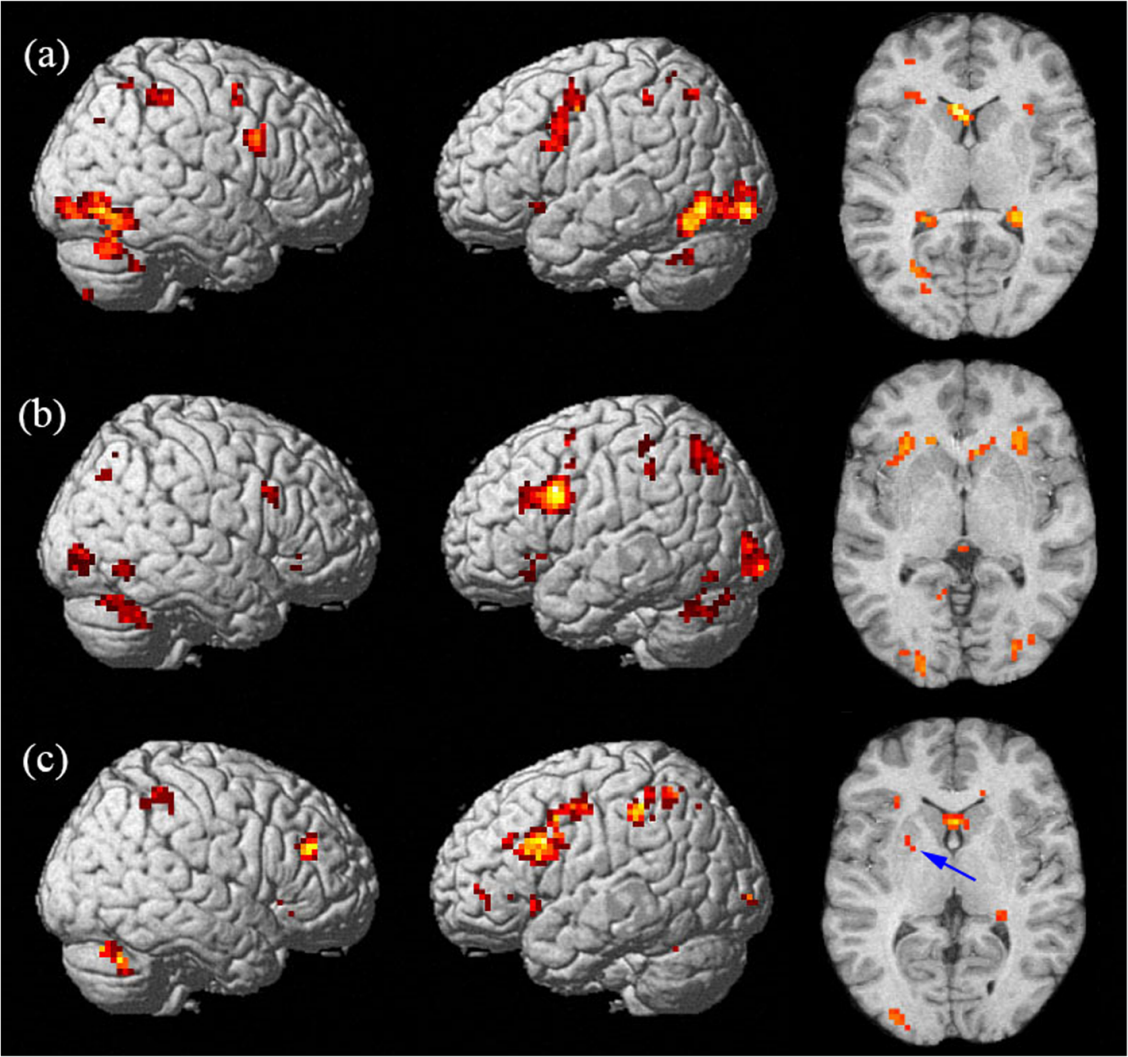
Brain activation maps superimposed onto stereotactically normalized T1-weighted image during three kinds of memory tests (*p* < 0.001, extent threshold *k* = 5). **a** Brain regions activated in short-memory test (STM). **b** Brain regions activated in long-term memory (LTM). **c** Brain regions activated in working memory (WM), with MrD of striatum pointed by blue arrow

The correct rates (correct response of total hits) were all above 75% for 19 subjects during STM task, 22 subjects during LTM tasks, and 21 subjects during WM tasks, and only the fMRI data for the subjects with correct rates over 75% were analyzed. The mean corrected rates of the subjects involved in data analyses were 96.05 ± 7.27%, 87.60 ± 6.49% and 85.71 ± 6.18% during STM, LTM and WM tasks, respectively. Table 1 lists the observed clusters showing and Fig. 2 shows the significant activation during the three different kinds of memory tests.

**Table 1:** Extent and intensity of activity in representative brain regions during different kinds of memory tests

Brain regions	STM				LTM				WM			
	BA	Coordinate (x, y, z)	KE	T value	BA	Coordinate (x, y, z)	KE	T value	BA	Coordinate (x, y, z)	KE	T value
Left frontal	6 8 47	0,11,52 − 48,8,41 − 30,32,-2	34 45 3	9.77 7.63 5.87	6 9 46 47	−3,14,46 − 48,13,30 − 48,30,21 − 18,29,-6	64 132 66 11	9.68 9.11 9.11 8.04	6 8 9 47	−45,2,44 0,17,49 −48,19,27 −48,17,-6	70 68 70 8	8.49 8.79 8.49 5.92
Right frontal	6 9 47	3,17,43 45,10,27 18,32,-2	34 26 5	9.77 8.19 6.49	9 47	39,13,32 33,29,-6	22 36	5.83 6.50	46	42,39,26	21	7.35
Left parietal	7 40	−24,-50,52 −36,-38,46	41 18	6.15 6.65	7 40	−27,-65,47 −39,− 36,40	72 29	7.38 7.85	7 40	-36,-42,38 −36,-44,44	72 145	8.65 8.65
Right parietal	7 40	27,-50,47 39,-47,52	64 19	7.33 3.98	7	27,-56,39	34	7.07	7 39 40	27,-45,38 30,-54,36 48,-59,39	9 9 29	5.41 5.36 7.71
Left occipital	18	−24,-94,-5	158	11.19	18 19	−21,-94,-5 −30,-59,-12	40 54	8.21 8.88	19 18 18	0,-15,-64 −6,-81,15 0,-24,-93	26 15 5	6.81 5.95 5.71
Right occipital	19	45,-67,-7	202	8.44	18 19	30,-82,2 39,-79,-4	19 19	6.09 6.09	18	24,-69,17	6	5.33
Left caudate	_	−12,21,13 −12,1,19 −24,6,0	17 23 10	8.01 6.90 5.55	_	−27,-35,5 −21,-10,25 −18,1,25	7 5 6	6.04 5.54 5.50	_	−3,21,4 −21,4,22	32 70	9.36 8.49
Right caudate	_	12,7,16 3,18,2	55 17	8.53 8.01	_	24,-34,10 18,1,22 9,23,-1	23 14 7	6.58 6.34 6.22	_	27,-38,7 21,-7,28	40 6	7.98 5.64

*STM* short-term memory, *LTM* long-term memory, *WM* working memory, *KE* the number of voxels for the cluster detected at *p* < 0.001); *BA* Brodmann’s Area

**Fig. 3:**
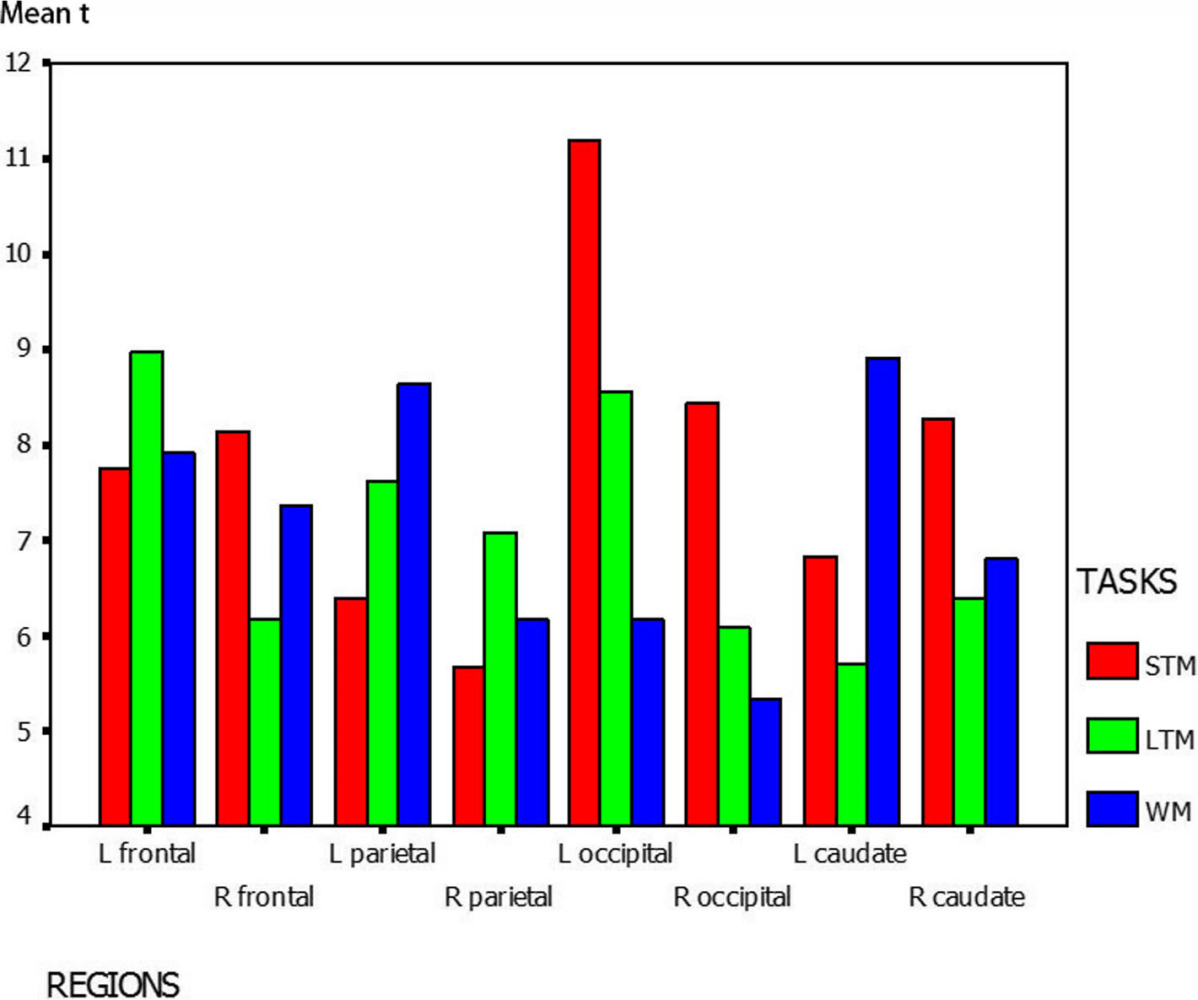
Mean *t*-value of each brain region activated during memory for digits. The graph shows the most activated structures located in the occipital cortex, left frontal cortex and left caudate in short-memory test (STM), long-term memory (LTM) and working memory (WM) for digits, respectively

**Fig. 4:**
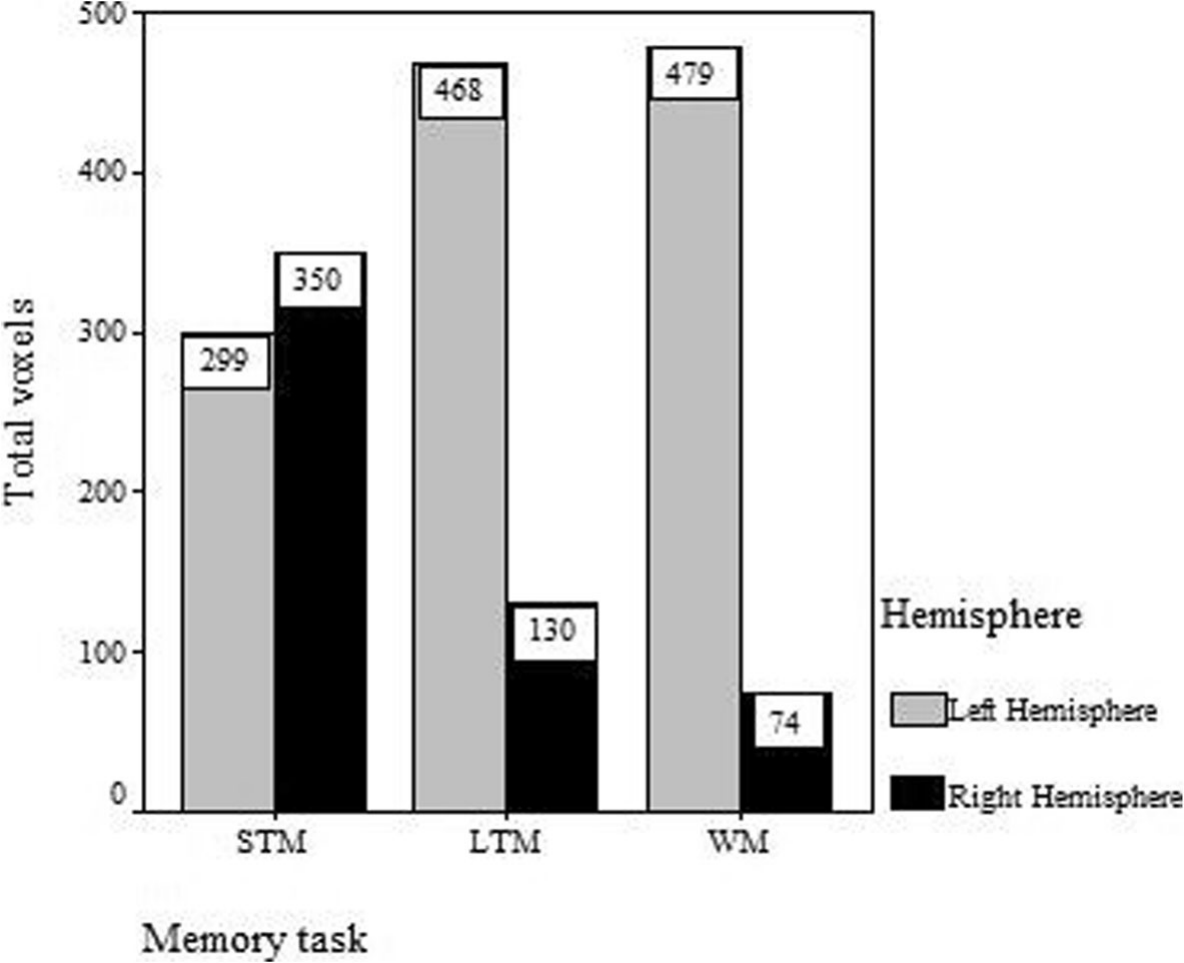
Comparison of total voxels between the left and right hemisphere during different kinds of memory tests. The bar plot indicates mild right hemispheric predominance in the short-term memory (SMT) test and significant left predominance in the long-term memory (LTM) and working memory (WM) tests based on the total voxel values

During the STM task, we found activation regions in the bilateral frontal lobe, parietal lobe, occipital lobe, caudate nucleus, inferior temporal gyrus, cingulate gyrus, putamen, caudate and cerebellum, as shown in Fig. 2a. The activated regions were identified as Brodmann’s Areas (BA) 6/8/9/47 in the frontal lobe, BA 7/40 in the parietal lobe and BA 18/19 in the occipital lobe. The left occipital lobe was most significantly activated and the right occipital lobe was also highly activated, as shown in Fig. 3. A mild right hemispheric predominance was found by comparing of total voxels between the left and right hemisphere during STM, as shown in Fig. 4.

During long-term digital memory task, BA 9/47 in the bilateral frontal lobe, BA 6/46 in the left frontal lobe, BA 7/40 in the bilateral parietal lobe, BA 18/19 in the bilateral occipital lobe, BA 37 in bilateral temporal, BA 32 in left cingulate gyrus, bilateral striatum and thalamus were activated (Fig. 2)b. The activation intensity of different brain areas was left predominant (Fig. 4). The activities in the left frontal and left occipital lobes were significantly higher than those in the right (Fig. 3).

During the WM task, we detected brain regions with significant activation in BA 6/8/9/47 in the left frontal lobe, BA 46 in the right frontal lobe, BA 7/40 in the bilateral parietal lobe, BA 39 in the right parietal lobe, bilateral hippocampus, cingulate gyrus, thalamus, caudatum, cerebellum and MrD of striatum (Fig. 2)c pointed by blue arrow) [15]. The left caudatum was activated most significantly and the activated brain areas in the frontal (Fig. 3) and parietal lobes were left predominant (Fig. 4).

## Discussions

It has been suggested that numbers have a multiple representational system and the brain regions involved in the memory task for digits are different from those for letters and objects [34]. An important question is which regions are recruited during the mental number processing and whether there are differential activations during the different cognitive manipulations of numbers. Current study aimed to detect brain activities when the healthy subject was visually stimulated by different patterns of numerical figures in his/her short- and long-term and working memories. Our results showed activation of different brain regions in these three kinds of memories, indicating that different kinds of memories rely on different neural correlates and mental processes.

In the STM test for numerical figures, the highest and largest activated area during STM for digits in current study were found in the occipital lobe (BA 18/19), which has been frequently reported as the crucial region involved in the visual STM [35]. It was indicated that the transient storage brain area for numbers was largely encoded by visual information, incompatible with the brain area involved in the general verbal STM which was located in the left lateral posterior temporal regions, supramarginal gyri, Broca area and dorsolateral premotor area [36]. STM for digits was the only memory examined with right hemispheric predominance (Fig. 4). This is in line with the notion that STM for numbers is encoded by visuospatial rather than phonological information according to Lycke’s studies, in which dominance in processing of phonological information was found in the left hemisphere while spatial information was predominately processed in the right hemisphere [37]. Similar result was observed in a previous fMRI study, which reported that subjects with high risk of Alzheimer’s disease showed an increased activity in bilateral visual occipital regions but their prefrontal regions could hardly be activated, and there was no significant neuropsychological difference between the high-risk and low-risk groups when performing a STM task [38]. We therefore speculate that short-term maintenance of numeric memory might be associated with more demanding cognitive resource relevant domains such as the prefrontal cortex. The bilateral parietal lobes especially the right parietal areas (BA 7/40) were reported to be involved in the retention of visual information by Munk and Todd [39, 40].

Crowder and Cowan, however, presented an opposite view that STM and LTM were different stages of the same representation, and the activated representations in LTM constituted all of STM. Evidence has indicated that the human medial temporal lobe (MTL) may not only be important for LTM consolidation but also for certain forms of STM [41]. Crowder argued that STM and LTM followed similar encoding processes and hence there was no reason to separate LTM and STM storage systems [42]. However, our results indicated that the active brain regions during STM for numeric digits were essentially different from those in LTM in the right frontal and bilateral occipital lobes. The most significant activity in the occipital lobes and the mild right lateral predominance in the activated brain regions implicated that STM information on numbers was mainly encoded by visuo-spatial representations while LTM by semantics.

Neuroimaging studies have identified a common network of brain regions consisting of the prefrontal and parietal cortices involved in different kinds of WM tasks [34]. In the WM test for numeric data, subjects were asked to refresh their short-term storage for the successively presented supplementary new numbers and compared them with the numbers which were shown before. The result showed that DLPFC (BA 8/9/46) and VLPFC (BA 47), bilateral parietal lobes (BA 7/40), bilateral hippocampus, cingulate gyrus, thalamus, caudatum and cerebellum were activated significantly. WM functions as a work-space in which recently acquired sensory information and information from LTM are processed for further action (e.g., calculation, decision-making). Although short-term storage was engaged in WM for numeric data, the brain regions activated in WM was different from those activated in STM. The caudate, prefrontal and parietal lobes were activated to the highest intensity with predominance on the left side in the current study. The activity pattern of the brain during WM for numeric data in this study was largely in line with previous studies on verbal WM, especially in the frontal lobe [21, 43]. According to the triple-code model proposed by Dehaene [19], magnitude was one of the most salient semantic representations of numbers. The neural correlates of number magnitude processing have been shown to be localized in the cortex around the intraparietal sulcus (IPS, BA 40) bilaterally [44]. Activities in the bilateral intraparietal sulcus were also found in this study. We speculated that those activities in the magnitude representation of two-digit numbers were required for memory and comparison by the subjects. Similar result was observed in Wood’s study [45]. There were common regions including the bilateral frontal, parietal and basal ganglia activated during WM and LTM memory task for numeric data. This observation was partly in accordance with Haarmann’s studies [46], which demonstrated an increase in neural synchrony between the prefrontal and posterior cortex and the enhanced activation of LTM representations of information held in WM. In other words, the LTM systems provide the necessary representational basis for WM. Hence there is no reason to posit specialized neural systems whose functions are limited to those of short-term storage and are distinct from LTM [47]. This is also consistent with the findings that the DLPFC contributed as the important link between WM controlled processes and LTM formation through its role in the strategic organization during encoding [48]. Lewis-Peacock and Postle in an fMRI study attributed the activity of prefrontal cortex (PFC) during LTM and WM tasks to the fact that the short-term retention of information was supported by the temporary reactivation of LTM representations [49].

Recently, there has been an increasing interest in the role of subcortical structures such as the caudate and the putamen. The MrD has been suggested to be involved in foot shock-avoiding memory [12] and an auditory WM for numeric data in an fMRI study [15]. We then proposed that the marginal division might play an important role in the digital WM by linking the limbic system with the cortex. In the present study, we also found that a small area in the left marginal division was activated during short-term digital memory as shown in horizontal section through the neostriatum (Fig. 3)a. Similar proposition was made by Chang, Crottaz-Herbette and Menon [50], whose connectivity analyses revealed an increased WM-load-dependent interaction of the left anterior caudate with the left posterior parietal, ventrolateral prefrontal and visual occipital cortex. In conclusion, the caudate has been proposed to link (relay) signals in distinct functional networks during WM task. Lesion study also indicated the important role of basal ganglia in digit processing [51]. The observation that the left caudate was highly activated in WM in the present study also concurs with our previous results. Therefore it would be a research focus for cognitive neuroscientists in the future to determine the biological functions of the subcortical structures and the complex neural network between the cortex and the subcortical regions in the memory process of the brain.

## Conclusions

Our study revealed the dissociation of activated brain regions during different patterns of memory for digits, regardless of the same stimuli, namely visual Arabic numbers. The primary processing and transient maintenance of STM for digits were located in the visual cortex and mainly encoded by visual representations. The right hemisphere was greatly involved. LTM was, however, encoded by semantic representations and left hemispheric predominant. The way in which numbers were encoded depended on the tasks in which they were involved. Similar behavior evidence was shown by Thevenot and Barrouillet [52]. The subcortical structures, bilateral caudate nuclei, were most highly activated in intensity in the WM tasks, which including multi-components such as recoding, storage and attention management. This arouses an increasing interest in subcortical structures, which might play an important role in linking the different cortical regions during the memory procedure. The neural network between the cortex and subcortical structures has also been proposed to be involved in the process of digital memory.

## Abbreviations

AD: Alzheimer’s disease
BA: Brodmann area
DLPFC: dorsolateral prefrontal cortex
fMRI: functional magnetic resonance imaging
LTM: long-term memory
MCI: Mild Cognitive Impairment
MrD: the marginal division of the striatum
SPM: statistics parametric mapping
STM: short-term memory
VLPFC: ventrolateral prefrontal cortex
WM: working memory
